# Recycling as a Key Enabler for Sustainable Additive Manufacturing of Polymer Composites: A Critical Perspective on Fused Filament Fabrication

**DOI:** 10.3390/polym15214219

**Published:** 2023-10-25

**Authors:** Antonella Sola, Adrian Trinchi

**Affiliations:** Advanced Materials and Processing, Manufacturing Business Unit, Commonwealth Scientific and Industrial Research Organisation (CSIRO), Clayton, Melbourne, VIC 3169, Australia

**Keywords:** additive manufacturing, fused filament fabrication, material extrusion, MEX, composite material, recycling, sustainability

## Abstract

Additive manufacturing (AM, aka 3D printing) is generally acknowledged as a “green” technology. However, its wider uptake in industry largely relies on the development of composite feedstock for imparting superior mechanical properties and bespoke functionality. Composite materials are especially needed in polymer AM, given the otherwise poor performance of most polymer parts in load-bearing applications. As a drawback, the shift from mono-material to composite feedstock may worsen the environmental footprint of polymer AM. This perspective aims to discuss this chasm between the advantage of embedding advanced functionality, and the disadvantage of causing harm to the environment. Fused filament fabrication (FFF, aka fused deposition modelling, FDM) is analysed here as a case study on account of its unparalleled popularity. FFF, which belongs to the material extrusion (MEX) family, is presently the most widespread polymer AM technique for industrial, educational, and recreational applications. On the one hand, the FFF of composite materials has already transitioned “from lab to fab” and finally to community, with far-reaching implications for its sustainability. On the other hand, feedstock materials for FFF are thermoplastic-based, and hence highly amenable to recycling. The literature shows that recycled thermoplastic materials such as poly(lactic acid) (PLA), acrylonitrile-butadiene-styrene (ABS), and polyethylene terephthalate (PET, or its glycol-modified form PETG) can be used for printing by FFF, and FFF printed objects can be recycled when they are at the end of life. Reinforcements/fillers can also be obtained from recycled materials, which may help valorise waste materials and by-products from a wide range of industries (for example, paper, food, furniture) and from agriculture. Increasing attention is being paid to the recovery of carbon fibres (for example, from aviation), and to the reuse of glass fibre-reinforced polymers (for example, from end-of-life wind turbines). Although technical challenges and economical constraints remain, the adoption of recycling strategies appears to be essential for limiting the environmental impact of composite feedstock in FFF by reducing the depletion of natural resources, cutting down the volume of waste materials, and mitigating the dependency on petrochemicals.

## 1. Introduction

Additive manufacturing (AM, aka 3D printing) has been hailed as the disruptive technology enabling the Industry 4.0 revolution [1], and laying the foundations for the advent of Industry 5.0, where humans and digital technology are mutually integrated [2]. One of the major challenges that materials scientists and engineers are presently called to face in the realm of AM consists in developing new printable feedstock materials that yield broad-ranging functional customisation [3]. Printable composite materials play a key role in this quest for improved mechanical performance (be it controlled stiffness, tensile or flexural strength, ductility, or fracture, fatigue, and creep resistance, depending on the targeted application) and embedded functionality (bioactivity, electromagnetic shielding ability, traceability, shape morphing and (self-)sensing ability, electrical and thermal conductivity, just to name a few). The technological maturity of composite feedstock is different for different AM methods, though [4]. While AM of ceramic matrix composites is still an emerging field [5,6], metal matrix composites are a research mainstay [7,8], with hundreds of papers being published every year. However, it is only with polymer AM that composite feedstock has transitioned “from lab to fab” [9]. The ever-increasing popularity of composite feedstock materials opens up new opportunities for polymer AM, but also creates new hurdles, especially in terms of sustainability.

With this perspective, the aim is to analyse the delicate nexus between AM, composite materials, and sustainability, and discuss the crucial role that recycling plays in overcoming the clash between the adoption of composite feedstock and the sustainability of 3D printing. In doing this, the focus is placed on polymer AM, and fused filament fabrication (FFF, aka fused deposition modeling, FDM) is assumed as a case study.

**Figure 1:**
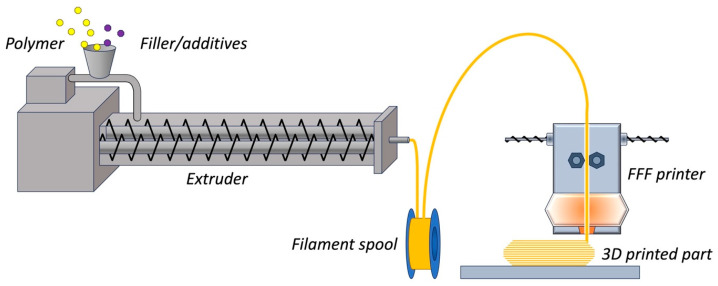
A thermoplastic filament produced by melt extrusion becomes the feedstock for FFF.

Strictly speaking, FFF belongs to the “Material extrusion” (MEX) family [10], as per ISO/ASTM 52900:2021 [11]. However, MEX is an umbrella-term that accounts for a broad range of technologies as diverse as FFF [12], direct ink writing [13], and bioprinting [14]. The peculiarity of FFF is that the feedstock is a thermoplastic filament, typically produced by melt extrusion. The filament is fed in a heated liquefier, melted, and pushed out from a nozzle while the print head moves according to a computer-controlled toolpath. Hereafter, the extrudates are referred to as “rasters”. Once the first layer has been deposited raster-by-raster on the base platform, a second layer is added on top of the previous one, and the process is then repeated until completion of the desired 3D geometry as illustrated in Figure 1.

**Figure 2:**
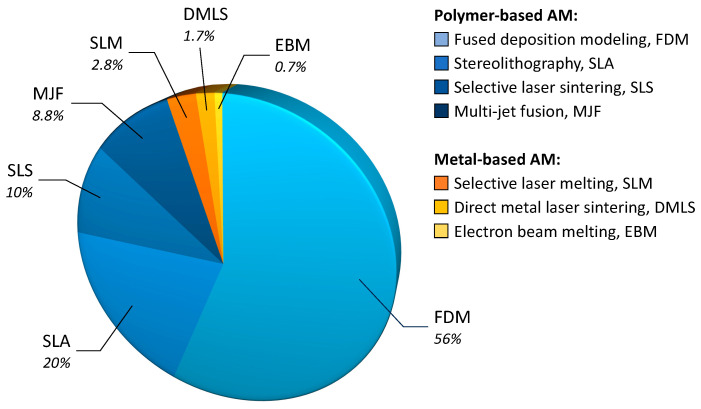
According to a survey conducted in 2023 [15], FDM (i.e., FFF) is being used by 56% of the businesses active in 3D printing.

The choice for FFF is dictated by many reasons. Firstly, according to recent statistics shown in Figure 2, FFF is by far the most widespread polymer AM technique, being used by more than 50% of the businesses in the 3D printing market [15]. FFF is affordable, versatile, and relatively simple [16,17]. As a result, FFF is extremely popular even outside “professional” settings. Many people have an FFF printer at home for hobby and do-it-yourself purposes, and FFF printers are being routinely run in schools, libraries, and small workshops [18,19,20,21,22,23,24].

Meanwhile, numerous composite filaments for FFF are already commercially available and easily accessible to everyone. As a result, the environmental footprint caused by the adoption of composite feedstock for FFF has transitioned from being a minor issue for R&D labs to becoming an open question with far-reaching implications for our society. While the progressive adoption of bio-based polymers and fillers may help reduce the dependency on fossil fuels, and the development of biodegradable materials may reduce the pressure on landfills, the disposal of end-of-life composite parts still represents a challenge due to the presence of heterogeneous phases. The primary objective of our review is to critically investigate the role that recycling plays in tackling this issue, while also considering methods for reducing the depletion of non-renewable resources and the energy consumption associated with the extraction and the synthesis of virgin raw materials.

## 2. The AM—Composite Materials—Sustainability Triangle

AM is gaining traction in science and industry for producing parts having bespoke geometry and highly intricated architecture. In a recent survey (February 2023), 76% of businesses (1035 respondents) declared that in 2022 they had used 3D printing for fabricating at least 10 parts or more in their production runs, up from 49% in 2021 [15]. This confirms that AM is gradually evolving from a rapid prototyping tool to an industrial manufacturing solution. The key advantage over conventional subtractive technologies like computer numerical control (CNC) machining is that 3D printed parts are built up by the progressive addition of material where it is needed, as opposed to the selective removal of material where it is not needed. In other words, this significantly reduces the buy-to-fly ratio, a parameter frequently adopted in aviation and aeronautics for quantifying the materials usage efficiency as the ratio between the amount of material needed to produce a part and the actual mass of the finished part itself [25]. In addition, 3D printing affords a more efficient use of feedstock materials, while also enabling the manufacture of more complicated structures. In a society increasingly aware of the depletion of natural resources being caused by overconsumption and waste, materials efficiency is one of the main reasons why AM is generally perceived as a “sustainable” fabrication method, although differences exist among diverse technologies and various feedstock materials [26].

Besides reducing materials waste, there are also other ways that AM may contribute towards a more sustainable economy. For example, 3D printed scaffolds, lattices, and topologically optimised parts minimize the amount of material being used while preserving their load-bearing capacity [27]. The adoption of lightweight components in airplanes, trucks, cars, and other vehicles is key to cutting down fuel consumption, with strategic advantages such as reducing our dependency on petrochemicals, and limiting environmental pollution, not to mention saving money [28,29]. In this regard, it is worth mentioning that even small weight reductions in transportation can make a sensible difference. For example, this is vital in aviation, to the point that Virgin Atlantic has estimated that cutting a pound (0.45 kg) in weight from every aircraft in its fleet would save 53,000 litres of fuel a year [30].

Being to a large extent a “digital technology”, AM is also subverting traditional supply chains. Instead of shipping components and spare parts from the producer to the end user (or middle person), design models and printing instructions can be shared on-line and then printed on-site. This avoids the need for moving goods [31]. AM also enables the fast delivery of spare parts, which can be conveniently printed on-demand, thus bringing down the delivery time and drastically reducing the space needed for stockpiling [32]. Another potential advantage comes from the reverse engineering and 3D printing of after-market parts [33]. Already common for legacy cars, this approach can be extended to industrial vehicles and machinery (provided that intellectual properties are protected), thus extending their lifespan beyond the failure of individual components that may become commercially unavailable over time.

The progressive diffusion of AM is backed by the continuous advancement of printing hardware and technologies. However, the portfolio of printing materials available in the marketplace is still very limited [34]. For example, it is estimated that the number of materials suitable for laser-based powder bed fusion (PBF-LB, aka selective laser melting, SLM), which is presently the leading technology for metal AM, is just around 50 [35]. This number narrows down even further to 10–12, if only market-ready materials are considered [36]. These figures are in stark contrast to conventional manufacturing, which harnesses more than 5500 metals and alloys [37]. In order to bridge this gap, research is being geared towards the development of new printable materials featuring improved mechanical performance and embedded functionality. While other strategies are also viable [3], the shift from conventional feedstock to composite materials appears to be the game changer, as the nature, relative amount, and spatial distribution of the constituent phases, including the matrix and one or more fillers and reinforcements, can be manipulated to target ideally any given service requirement [38].

The transition to composite materials is especially attractive for plastic-based AM technologies like FFF, since the addition of fibres and other reinforcements remediates the otherwise poor mechanical strength of the polymer matrix [39]. For example, polymer AM is very well suited to producing musical instruments with excellent sound quality [40]. To this end, chopped carbon fibres can be added in order to increase the stiffness and creep resistance of poly(lactic acid) (PLA), one of the most widespread polymer feedstocks for FFF. Carbon fibre-reinforced PLA filaments are thus ideal for printing music instruments such as violins, where they can resist the continuous structural load being generated by understrings [41]. Many industries are routinely producing their metal-forming dies and tools by FFF. However, replacing conventional tool steel dies has only been possible by leveraging the stiffness and toughness of carbon fibre-reinforced plastic filaments [42,43]. Another example of what composites can do for FFF in industry is given by the fast fabrication of end-of-arm tools, which are the “hands” at the end of robotic arms for industrial automation. End-of-arm tooling should be conformal to the object that the robot is intended to manipulate. Naturally, end-of-arm tools can be rapidly prototyped and iterated by FFF for a fraction of the cost of traditional tooling. However, conventional plastic feedstock would be unsuitable, since these tools need to be lightweight, strong enough to lift heavy parts, and durable enough to withstand thousands of cycles. This unique combination of properties is attainable by polymer-matrix composites, which also offer the additional advantage of being resistant to corrosive machining fluids [44].

Among others, these are just a few case studies that demonstrate the success of commercial composite feedstock for FFF. Notably, these examples have all been taken from the website of filament producers and suppliers, and not from the archival literature. However, FFF of composite materials also comes with scientific and technical challenges [45]. One of the main hurdles associated with composite materials is the increased environmental burden, because adding a filler necessitates additional processing [45] and makes the disposal of supports, scraps and end-of-life parts challenging [46,47]. Papers directly comparing the environmental impacts of mono-material and composite feedstocks in FFF are still very rare. For example, a cradle-to-gate life cycle assessment (i.e., from raw materials’ extraction to printing) revealed that if olive wood scrap is added to PLA in order to print an ornamental object of a given volume, the total environmental impact decreases (by up to 10.2% with 20% of wood). However, this advantage is primarily afforded by the fact that in the composite feedstock, a certain percentage of olive wood replaces PLA, where the production of PLA granulate is the main contributor to global warming potential and to abiotic depletion—fossil fuel, whilst the impact of wood scrap (as a natural by-product of an artisanal workshop) is negligible [48]. Meanwhile, the energy consumption for extruding and printing notably increases over neat PLA due to the impaired melt rheology of the polymer matrix. It is also worth noting that a cradle-to-gate assessment does not account for the usage of the printed composite parts. For example, it was also observed that flexural stiffness, strength and deformation at break were all nearly halved by the addition of wood (by 20%), likely because the composite parts had not been printed with optimised parameters, or because the wood scrap had not been surface-treated prior to melt compounding with PLA [48]. This is an important point because the environmental friendliness of FFF depends on the specific application and the operating loads that the printed parts must withstand [49]. Also, a cradle-to-gate analysis does not consider the end-of-life stage of a product, and this is a crucial point, as disposal may be difficult for composite parts that contain heterogeneous materials [50].

## 3. Critical Considerations about “Sustainability” and “Sustainable Materials”

To the authors’ minds, wondering if additively manufactured composites are “sustainable materials” appears to be an ill-posed question. Firstly, it is worth mentioning that at present, the concept of “sustainable material” remains intuitive, but not quantifiable. Back in 1987, the United Nations Brundtland Commission defined sustainability as “meeting the needs of the present without compromising the ability of future generations to meet their own needs” [51]. With this statement (which is reported in Table 1 with other recycling-related keywords [33,51,52,53,54,55,56,57,58,59,60,61], where the listed reference(s) for each term redirect to the source(s) of the corresponding definition), the United States clearly established a link between “sustainability”, on the one side, and societal responsibility towards future generations, on the other side, while still recognising the importance of satisfying present needs. Conceivably, this can be pursued (also) through the wise sourcing and management of materials. According to Rutgers, Centre for Sustainable Materials, “Sustainable materials are materials used throughout our consumer and industrial economy that can be produced in required volumes without depleting non-renewable resources and without disrupting the established steady-state equilibrium of the environment and key natural resource systems” [52]. It may be worth adding that sustainable materials should be easily reprocessed or recycled, thus reducing the need for raw materials [62]. In the discussion about the sustainability of producing composite materials by AM, McCarthy and Brabazon [33] state that “Sustainability is the potential for something to continue indefinitely”. Ultimately, the idea of “sustainable materials” revolves on circularity, be it enabled by the transition to renewable sources, or by the promotion of continual reuse. Yet, there is no one clear definition for “sustainable material”.

Quantitative information can be achieved through a life cycle assessment (LCA), which is a standardised method of calculating the environmental impact of a given product [63]. During the LCA, environmental effects produced through the product’s life are expressed into actionable numbers called “impact categories” [64]. In order to make the data more manageable, the impact categories can be aggregated in a single metric, such as the Environmental Cost Indicator (ECI). This is very practical for comparative purposes [65]. For example, the efficacy of a circular initiative can be estimated through the drop in the ECI associated with the reduction in energy consumption, gas emissions, or materials waste enabled by the initiative. Otherwise, the Material Circularity Indicator introduced by the Ellen MacArthur Foundation measures how restorative the material flows of a given material are [58,59]. However, we do not have a single parameter that just says if a given material is “sustainable” or not.

Meanwhile, it should be mentioned that new materials, and especially composites, are developed in order to address specific needs, and somehow the “sustainability” of a material may also depend on its intended application. For example, the “sustainability” of a composite made of continuous carbon fibre-reinforced epoxy resin may appear questionable. If compared to the neat polymer, the composite requires energy-intensive manufacturing (pultrusion, for example [66,67]), and may also need special chemicals (a sizing for improving the adhesion to the polymer matrix, for instance [68]). Moreover, recycling is extremely difficult and expensive, as the thermoset matrix must be broken down thermally (pyrolysis) or chemically (solvolysis) to recover the precious carbon fibres for reuse [69]. However, if the fibre-reinforced epoxy was used for replacing steel in vehicle bodies, this would lead to substantial weight savings, and this, in its turn, would increase fuel efficiency and hence sustainability [70,71]. Somehow, the environmental benefits associated with the final application of the composite would offset the increased environmental footprint associated with processing and disposing.

Given all the above, if accepting the assumption that composite materials are needed to unlock the full potential of AM, to the authors’ mind it seems more profitable wondering the following: how can we minimise our environmental footprint as we pursue the AM of composite materials? This also resonates closely with the definition provided by the United Nations, which already acknowledged the relationship existing between sustainability and the necessity of meeting the needs of the present while reducing our environmental impact.

## 4. Recycling as a Key Enabler for Sustainable FFF of Polymer Composites

When it comes to FFF, an analysis of the literature reveals that attention is being paid to two main strategies for reducing the environmental burden associated with composite materials, namely turning to renewable resources, and recycling. In principle, for a composite material to be considered “fully green”, both the constituent phases, i.e., matrix and reinforcement/filler, should come from renewable resources or be recycled [72].

To the best of the authors’ knowledge, FFF is one of the few AM techniques for which polymer feedstocks derived from renewable resources are already available in the marketplace. Although the main reason for its popularity is certainly the ease of printing, PLA also takes advantage from being bio-based, since it is derived from plant sources like cellulose, starch, corn, or from fish and kitchen waste [73]. As a result, PLA is widely promoted as a sustainable feedstock for FFF, which helps us break free from petrochemicals [74]. In this regard, it is worth mentioning that petrochemicals and their derivatives absorb an increasing amount of the world’s oil and gas (approximately 14% in 2018 [75]) and are becoming the largest driver of global oil demand, ahead of road freight, aviation, and transportation [75,76]. Unlike most commodity thermoplastics like polyethylene (PE) and polypropylene (PP), PLA is also biodegradable. While PLA degrades within weeks under industrial composting conditions (62 ± 4 °C, >60% relative humidity), it does not degrade as easily in natural environments, to the point that degradation can take up to a year in soil or domestic composters with a temperature of 20 °C [77,78]. However, biodegradation can be sped up through the molecular design of PLA, for example through the copolymerisation of lactide (LA, which is the basic unit of PLA macromolecules) with other monomers that establish chemical bonds more susceptible to hydrolysis [77]. Other polymers obtained from renewable resources, such as biomass-derived polymers [79], and polyhydroxyalkanoates [80], including poly(hydroxybutyrate) [81], are printable by FFF [82], but just a few of them are market-ready [83]. Conversely, it is worth mentioning that some plastics commonly used in FFF are biodegradable, in spite of being petroleum-based. This is the case, for example, with PET, which can be biodegraded by *Streptomyces* species, and with polystyrene (mainly, high impact polystyrene, HIPS), which can be biodegraded by *Bacillus* spp. and *Pseudomonas* spp. [84,85]. Interestingly, polyvinyl alcohol (PVA), which is water soluble, is also biodegradable in the presence of suitably acclimated microorganisms [86].

As for the reinforcement, nearly any vegetable filler possible has been tested for FFF, from continuous fibres [87,88,89,90] to wood flour [91] to nanocellulose [92,93]. Short fibres have been obtained from jute [94,95], kenaf [96], bamboo [97,98], flax [97], hemp and harakeke [99,100], banana and other palm trees [101,102], agave [103], and bagasse [104], just to name a few. Since they come from renewable resources and are also biodegradable, vegetable fillers are commonly deemed as green and sustainable alternatives to synthetic ones, like glass and carbon fibres [105]. Also, wherever possible they are recovered from waste or by-products of other primary agro-industrial activities [106,107], like wood flour being sourced from furniture waste [108]. However, it is worth mentioning that vegetable fillers often need to be extensively treated prior to melt-compounding with the polymer matrix. They must be thoroughly pre-dried before processing to avoid the release of water vapour at high temperature [109], and they also necessitate mechanical and/or chemical treatments such as calendaring, stretching, and sizing in order to improve their durability and ameliorate the interface bonding with the polymer matrix [110,111]. Moreover, being strongly hydrophilic, vegetable fibres may favour moisture uptake, which rapidly impairs the structural reliability and shortens the lifetime of the 3D printed composite [112,113]. Fillers of animal origin such as feather fibres and bone powder can also be employed in polymer matrix composites for FFF [114,115,116]. However, they are mainly sourced from by-products and waste of the food industry. As such, they can be classified as recycled materials. Likewise, clays [117], talc [118], gypsum [119], and other minerals are of potential interest as the reinforcing phase in FFF. A quick search online reveals that marble-filled filaments are already commercially available for simulating the appearance of “real” marble objects, while filaments containing powered chalk are popular amongst hobbyists because they produce parts with a smooth surface finish. However, although this point remains a subject of debate, it is worth noting that, in spite of being “natural” (as opposed to human-made) fillers, minerals are non-renewable [120]. Again, much as vegetable fibres and animal fillers, minerals for FFF are also primarily recycled from other businesses [121,122].

Ultimately, turning to renewable materials is certainly advantageous for limiting the depletion of natural resources, but dramatically narrows down the range of ingredient materials that can be used for producing printable composites. As valuable as they are for helping us break free from petrochemicals and save other finite natural resources, a handful of renewable polymers combined with vegetable fillers and fibres cannot satisfy the appetite for bespoke functionality that is the driving force for developing new printable polymer matrix composites.

**Figure 3:**
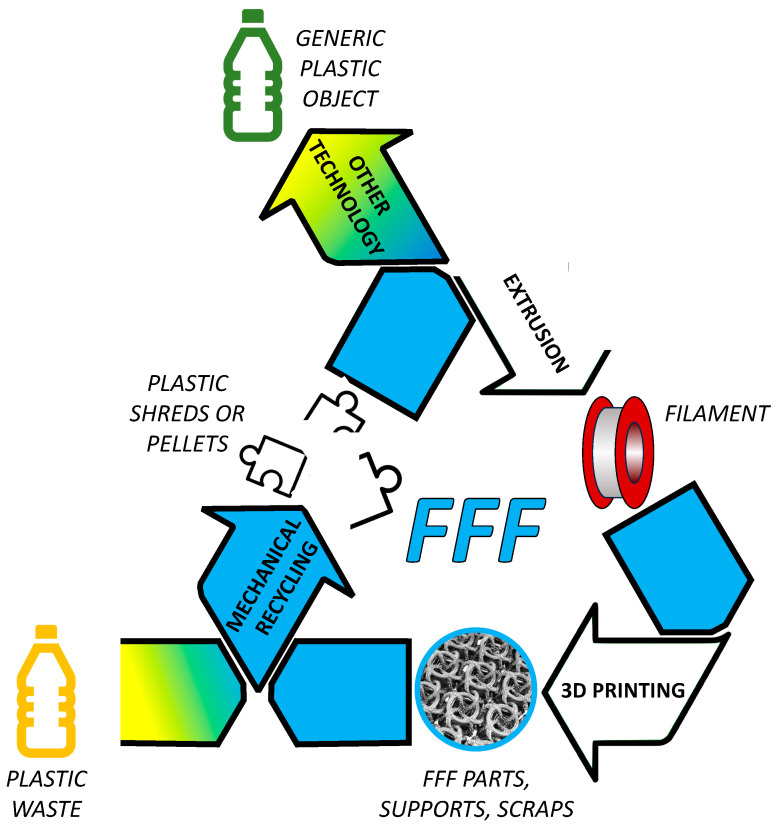
Integration between plastic recycling and FFF.

Recycling may be regarded as a more versatile approach, especially when it comes to FFF. In fact, a basic distinction should be drawn here between FFF, which uses thermoplastic polymers and does not entail permanent crosslinking, and other polymer-based AM methods like vat photopolymerisation that require instead curable resins or inks [123]. In terms of sustainability, working with thermoplastic feedstock certainly represents a strategic advantage of FFF [124]. As schematically presented in Figure 3, the advantages are actually twofold, meaning that recycled thermoplastic polymers can be used for producing printable filaments [125], and, in principle, FFF parts can be recycled like any other plastic item [126]. Even FFF parts and scraps can be crushed and re-extruded for producing new printable filaments, which closes the loop and leads to establishing a circular economy for 3D printing [127,128,129,130]. Meanwhile, recycled materials can also be used as the reinforcement/filler [16,131,132]—and, in this respect, recycling may offer key advantages in terms of sustainability not just for FFF, but for any plastic AM technology and, even more broadly, for any AM technology, regardless of the matrix composition. Of course, the two strategies, namely, opting for renewable resources and recycling, can be combined, for example, through the recycling of composite parts originally produced from renewable materials.

Finally, recycling is surely the game changer for enabling the sustainable development of composite feedstock for FFF, but upon looking more closely, challenges and obstacles still exist.

## 5. Recycling for Producing Composite Feedstock for FFF

### 5.1. Thermoplastic Matrix

According to recent estimates, around 8.3 billion metric tons of plastic have been produced worldwide since 1950. This has become a major cause of pollution, since around 80% of plastic waste has ended up in landfills or dumped and dispersed in the environment [133,134]. Although recycling plastic materials as the feedstock in FFF can only target a very minor fraction of this plastic waste, still it contributes to solving the issue. Also, recycling FFF parts can help reduce the impact of 3D printing on the environment [135]. Notably, plastic recycling in FFF is more than a research topic, it is an industrial reality, in that several 3D printing filaments derived from recycled plastic waste are already in the market [125].

Owing to the thermoplastic nature of FFF feedstock, the recycling workflow is theoretically very simple, since collected plastic waste can be shredded, extruded, and printed again [136]. However, this apparent simplicity hides several difficulties [137].

Firstly, waste streams should have consistent composition to make the process repeatable. Besides meeting the consumer’s expectations in terms of printed part’s quality and performance, filaments with tightly controlled properties will reduce the number of failed printing jobs, and thus the volume of plastic scraps.

For a given waste stream, the volume of material available for recycling must be sufficient for scaling up. Distributed recycling via additive manufacturing (DRAM) is gaining momentum for the in-situ management of relatively small volumes of plastic waste by means of mechanical recycling and 3D printing [138]. However, it is worth mentioning that a single commercial spool commonly ranges between 500 g and 1 kg, and scalability may represent a challenge, especially if the quality must be consistent across different filaments’ batches [139,140].

Aside from being clean and readily available, plastic waste must be affordable. The energy required for recycling is generally lower if compared to the energy required for extracting the raw materials and producing the virgin plastic [141]. However, recycling is labour intensive, and hence very costly, especially if the quality and composition of the recycled material must be tightly controlled. In order for recycling to be a viable option, the costs for collecting, sorting, washing and pre-treating, crushing or pelletising, and shipping (if required), must not exceed the cost for purchasing the virgin plastic.

In this workflow, “sorting” likely represents one of the most complicated steps. Presently, sorting technologies produce a limited fraction (generally, not exceeding 60–65%) of mono-streams, while the rest remains as mixed plastic waste [142]. However, plastics are rarely used on their own. Plastic objects are indeed multi-component systems [143], where different polymers can be mixed together in different ratio (polymer blending) to achieve the desired functionality [144], and combined with a multitude of plasticisers and dies. Other additives can also be introduced for tuning the mechanical and ancillary properties of the base polymers [145]. Finally, the composition is likely heterogeneous even within individual mono-streams. Sometimes, the co-existence of different plastics in the same stream may offer some advantages. For instance, as part of a Colombian project that aimed to fabricate simple assistive devices for disabled people, recycled filaments from collected polyethylene terephthalate (PET) bottles were mechanically reinforced by the presence (5 wt.%) of high-density polyethylene, which may come in as an impurity from caps and rings. However, achieving strong inter-layer bonding in the printed parts was troublesome, due to recycled PET being very sensitive to the applied processing conditions [146]. Moreover, the presence of impurities or thermally labile substances may be particularly problematic in FFF, since they release gases and volatile compounds when heated upon printing. Entrapped bubbles coalesce into micro-voids, which may become crack initiation points, and cause surface roughness and irregularity in the printed part [130,147]. Also, the release of chemicals, often associated with fine particulate, represents a risk for operators to be exposed to hazardous irritants and even to potential carcinogens [22].

One of the main pitfalls of mechanical recycling is that repeated thermo-mechanical processing may damage the molecular structure of thermoplastic materials and also trigger uncontrolled crystallisation [129]. The combined action of thermal and mechanical loads is widely reported to provoke polymer chain scission [144,148,149,150]. As the average molecular weight decreases, the rheological behaviour changes [151], and the printability worsens with it [152]. When FFF parts are crushed and recycled for printing again, the mechanical strength of the printed parts can be retained over a few re-processing cycles, typically 1 or 2, but is then expected to drop [135,153,154,155,156,157]. In an experimental study that investigated the potential of close-looped recycling of PLA, commercial granules were extruded and printed. Then, the printed parts were shredded, and re-extruded for repeated printing cycles. PLA could only be reprocessed two times, as the third printing cycle failed due to the reduced melt viscosity associated with excessive molecular degradation. Notably, in spite of the reduced molecular weight of PLA, only minor changes were observed in the mechanical properties. Meanwhile, an LCA that, for a given mass of printed material, the environmental impacts associated with close-looped recycling were lower than those of incineration or landfilling. In turn, the environmental burden of incineration was lower than that of landfilling, owing to the recovery of the calorific value embodied in the PLA parts [135].

In the meantime, shorter molecules can re-arrange themselves into ordered structures more easily [158], and this may spur crystallisation phenomena. While crystallisation is often accompanied by an increase in stiffness, the polymer may also become more brittle. Further to this, polymers that crystallise rapidly upon cooling may not have enough time for healing the raster-raster interface, and this is a major reason for premature failure in FFF parts [12]. Crystallisation is also accompanied by a noticeable change in specific volume, which makes the part shrink and deform, to the point that it may easily peel off from the baseplate while printing [159].

Fillers are frequently added to recycled plastic matrices to mitigate the adverse effect of repeated thermo-mechanical processing and help restore some properties, such as the tensile stiffness and strength of the printed parts [144]. For example, waste bakelite and ceramic particles (silicon carbide and aluminium oxide) have been added to recycled ABS [160,161], gypsum to recycled PP [119], and chopped glass fibres to blends of recycled PET and recycled high-density polyethylene (HDPE) [162]. Quite often, wood is explored to obtain “fully green” composites, with the advantage of improving the thermal stability of recycled PLA [153]. However, though apparently paradoxical, the addition of fillers in composite materials may exacerbate some printing-related issues [38], as fillers may act as “pinning points” that restrain the chain mobility of the polymer matrix with adverse consequences on inter-raster and inter-layer healing [163], and may also act as nucleating agents, thus promoting crystallisation [164,165]. Depending on the system’s composition, fillers may even catalyse the degradation of the polymer matrix through hydrolysis and thermolysis phenomena [166]. For instance, hydroxyl groups anchored to the filler’s surface are known to interact with the ester bond of PLA and induce polymer chain scission [167]. Conceivably, the presence of fillers also adds complexity to recycling.

### 5.2. Fillers

Although still uncommon in the marketplace, fillers derived from waste and recycled materials are gaining momentum in the literature. As previously mentioned, while offering key advantages towards achieving FFF parts with embedded functionality, the transition from neat thermoplastic filaments to composite ones poses numerous challenges due to the multi-facetted effect of the filler on the printability of the polymer matrix [12,38,45,168]. However, interestingly, using recycled fillers instead of virgin ones should not cause any additional issues, provided that the recycled powders or fibres match the physical characteristics (size and shape, for instance) and thermomechanical properties (stiffness and thermal stability, among others) of the virgin counterparts.

The literature presents an extraordinary variety of recycled fillers, as diverse as artisanal ceramic waste [169] and ground tire rubber [170,171], exhausted metal powder from other manufacturing systems and printing technologies [172], paper products [173,174] and textiles [175], and a number of waste products from agriculture and the food industry, such as crushed crab shells [176,177], almond skin powder [178,179], ground walnut [180,181] and cocoa bean [182] shells, powdered peach kernels [183], corn [184] and rice [185] husk, and pyrolyzed soy hulls [186]. Reclaimed carbon fibres can also be harnessed as the reinforcement in FFF composites [131,149,187,188,189,190,191,192,193,194,195]. The recovery of carbon fibres for 3D printing by FFF is gaining attention owing to the high cost of these fillers, and to the polluting consequences of their disposal [196,197].

Each filler comes with specific recycling challenges depending on its composition and origin. For example, (organic) wood flour obtained from pulverised disposable chopsticks [198] and (inorganic) glass fibres reclaimed from wind turbine blades [17] are likely to follow different recycling procedures and require different compounding strategies for producing printable filaments. As a result, generalising is not possible. Nonetheless, some considerations hold true irrespective of the filler’s nature. Firstly, much like recycled polymers, recycled fillers should be consistent and readily available in large volumes, which is why recycled fillers are preferentially sourced from commercial and industrial streams [199]. Their size and shape should be tightly controlled. While filaments come in two standard diameters of either 1.75 mm or 2.85 mm, the print nozzle is in fact much smaller, with the commonest diameter being 400 µm [12]. Fillers should not cause clogging, and, for this reason, reinforcements are typically micro- or nano-sized (printing continuous fibre-reinforced parts is also feasible but requires specialised printers [200,201,202]). On the other hand, the finer the filler, the higher the likelihood of agglomeration [203], which will increase the risk of blocking the nozzle, and reduce the specific surface area available for matrix-filler interactions. Another potential issue associated with recycling consists in damaging the filler. For example, cenospheres, which are hollow spherical particles coming as by-products of coal combustion [204], may be added to the polymer for 3D printing syntactic foams [205]. However, cenospheres are brittle, and hence likely to break while extruding the filament and printing. The fragments are very sharp, and much harder and stiffer than the polymer matrix. Broken cenospheres are thus expected to cause local stress concentration, and trigger cracks [206]. Likewise, fibre breakage may occur due to the collision of the fibres with other fibres, with the matrix, and with the internal walls of both the extruder and the printer [207,208,209,210,211]. As the filler loading increases, the number of fibre-fibre interactions also increases, and this worsens fibre breakage [149,187,212,213]. Since the fibre diameter remains unaffected, the aspect ratio (which is the fibre length-to-diameter ratio) decreases, and this is predicted to reduce the strength of the composite due to impaired matrix-fibre load transfer [214,215]. In recycling, alleviating fibre breakup is thus imperative, and, wherever possible, the fibre length should remain above the critical length, which is the minimum fibre length required for the fibres to be stressed to their ultimate strength [132].

Meanwhile, recycling may damage the thermo-mechanical properties of the reinforcement. Although this may be more obvious for vegetable fibres owing to their poor thermal stability [196,216], inorganic fillers may also be affected by the temperatures and chemicals required for recycling [132,197,217,218]. For example, the tensile strength of glass fibres may be reduced by 80–90% after thermal recovery [219], and similar deleterious effects have been reported for silica treated above 200 °C [220] and basalt fibres treated above 180 °C [221]. In spite of their thermal stability, carbon fibres are also prone to oxidation if thermally treated in air [222], with the ultimate heat-resistant temperature being around 400 °C [223]. Indicatively, depending on the recycling method, recovered carbon fibres retain approximately 70–90% of their original strength [224]. However, it is worth noting that not all processing-induced changes are disadvantageous [132]. For example, it has been reported that removing the sizing of carbon fibres using pyrolysis and solvolysis methods modifies the surface morphology of the fibres, and this improves the interfacial shear strength when the fibres are dispersed in a polymer matrix [225].

In conclusion, although the purity of the material can be preserved to a large extent by implementing accurate and highly selective sorting procedures, this inevitably increases the cost of recycling. On the other hand, the polymer degradation and the functionality loss of the filler/reinforcement commonly associated with recycling can be mitigated, but not avoided. This requires striking a balance between performance, sustainability, and cost. However, the sweet spot significantly differs from one composite material to another, and finding a trade-off requires a combined technological-environmental-economic assessment, which should also take into account existing laws and funding schemes.

## 6. Recycling and FFF of Composite Materials: New Trends and Future Opportunities

A survey of the literature reveals that, on account of its practicality, thermo-mechanical recycling is the preferred option for recycling the polymer matrix, as thermoplastic materials (after shredding, if required) can be melted and reprocessed, although this may cause some degree of degradation. Reasonably, different recycling strategies are required for different fillers, depending on their composition (hemp fibres vs. glass fibres, for example), their morphology (short carbon fibres vs. continuous carbon fibres, for example), and their economic value. In this regard, carbon fibres have received much attention in the literature, with recycling being generally credited as a more sustainable practice than landfilling. Specifically, close-looped recycling of carbon fibres helps mitigate the environmental impacts in FFF because the recovered fibres avoid the production of virgin fibres for producing composite parts. For example, encouraging results are emerging regarding the environmental sustainability of close-looped recycling of carbon fibres in FFF through solvolysis. However, this recovery process is likely to cause fibres to degrade, and the functionality loss, which largely depends on the specific recovery process, will ultimately dictate the load-bearing capacity and the lifetime of the printed object [50].

Besides receiving recycled plastics and recycled fillers as the input materials, FFF is emerging as a viable means to mechanically recycle composite materials without any prior treatment for separating the matrix and the reinforcement. For example, functional composite filaments can be produced from multi-layer aluminium-plastic packaging (MLAPP). MLAPP is widespread for containing and protecting perishable goods, like milk and beauty face masks [226]. In order to maximise its sealing barrier performance, MLAPP combines multiple layers made of thermoplastic polymers (around 80–85 wt.%) and aluminium (remaining 15–20 wt.%). MLAPP is typically single-use, and recycling is extremely difficult because of the high binding strength (120 to 160 MPa) between the laminated layers. In addition to being economically unfeasible, conventional separation methods require large volumes of solvents and complex multi-step procedures that lead to high CO_2_-equivalent emissions [227,228]. A possible way out consists in pulverising MLAPP waste without any separation, for example by solid-state shear milling, thus obtaining a composite powder that combines polymer and oxidised aluminium particles. The composite powder can then be extruded into a printable filament that features electrical insulation properties due to the presence of oxidised aluminium. Moreover, if need be, additional functional fillers can be introduced while extruding the filament, such as expandable graphite (EG) that imparts high thermal conductivity [228]. A similar strategy has been successfully implemented for recycling multi-component meal-ready-to-eat (MRE) pouches in use with the United States (U.S.) Army as a proof of concept of rapid materiel resupply for 3D printing in the field [229].

Interestingly, experimental evidence exists highlighting that objects made of thermoset resin such as Bakelite can be milled and employed as the reinforcing phase or functional filler in composite filaments for FFF [230,231]. This paves the way for a new recycling strategy for thermoset composites that does not rely on fibre recovery. The concept has also been demonstrated, for instance, with melamine-particleboard-paper impregnated with phenolic resin, which is commonly found in wood-based waste from the furniture industry [108].

In terms of sustainability, one of the main objections being moved against the adoption of composite feedstocks in FFF is that multi-phase materials are more difficult to recycle than neat polymers. However, in principle, composite parts printed by FFF are recyclable like any other thermoplastic matrix composite [132]. In principle, closed-loop recycling can be accomplished by crushing, extruding, and printing again the composite parts, as well as the composite waste coming from failed print jobs and support structures. On the other hand, many authors in the literature are already used to extrude, chop down their composite filament, and extrude it again (sometimes repeatedly) in order to improve the homogeneity of the filler distribution and remove potential bubbles [232]. Otherwise, open-loop recycling is also doable, since FFF composites can be mechanically treated to become the feedstock for other thermoplastic-based manufacturing methods such as compression moulding [46]. Either way, as previously mentioned, polymers are likely to degrade because of thermal and mechanical re-processing. However, their workability and mechanical properties can be restored with proper additives, such as chain extenders [148,233]. Recycled materials can also be supplemented with virgin feedstock. Alternatively, new experimental findings prove that conventional thermoplastic materials such as acrylonitrile-butadiene-styrene (ABS, which is likely the most widespread plastic for FFF together with PLA, as well as the key ingredient of classical LEGO bricks [234,235]), can be changed into a vitrimer, which is a crosslinked system based on thermally reversible covalent bonds. Since the dynamic covalent cross-links can be opened and restored during reprocessing cycles, the strength and characteristic temperatures of the virgin vitrimer can be preserved through repeated mechanical recycling [236]. Meanwhile, new strategies are also being put forward for recovering and remanufacturing high-value reinforcements like continuous carbon fibres, which can be extracted from FFF composite parts after locally remelting the polymer matrix with a hot air gun that moves backwards along the original printing toolpath [237].

Special attention should be paid to nanocomposites, which are becoming increasingly popular in research, and also in commercial products, including feedstock for FFF [238,239,240]. In terms of sustainability, one of the main advantages of nanocomposites is the so called “nano-effect”, whereby a substantial enhancement in properties, such as mechanical, thermal, electrical, or chemical, can be achieved by small additions of nanofillers [241]. In other terms, nanocomposites often require smaller amounts of fillers while still outperforming conventional composite systems. Besides saving critical materials, when used in FFF, nanocomposites are less prone to cause blockages in the printhead because filler agglomerates are orders of magnitude smaller than those of conventional fillers, which reduces the volume of print scraps that should be disposed of. Moreover, unless nanofillers possess a high aspect ratio (which is the case, for example, of carbon nanotubes [242]), their size is only marginally affected by repeated thermo-mechanical processing. However, the drawback is that nanofillers may pose safety issues, with their toxicity being amplified by their vast specific surface area. Also, loose nanofillers may be inhaled easily. After that, they are known to affect the respiratory tract, or even interstitialise in the lungs [243]. Once compounded in the polymer matrix, nanofillers become isolated and thus theoretically harmless. Nonetheless, they may be released to the environment while producing the filament, especially during the feeding step. Moreover, nanocomposites may experience wear, weathering, or biodegradation processes in service, and this leads to fragments at the nano- and micro-scale being dispersed in the environment [244]. There are also many uncertainties related to the safe disposal of nanocomposites. If simply disposed of in landfills, nanocomposites will likely degrade due to the combined action of light, heat, moisture, liquids of variable pH and ionic strengths, microorganisms, and other physical, chemical, and mechanical factors. Incineration may burn carbonaceous nanofillers off, but not thermally stable ones, such as ceramic or metal nanoparticles, which would require appropriate control procedures for entrapment [245]. However, experimental findings suggest that mechanical recycling of thermoplastic matrix nanocomposites, if properly conducted, does not generate more airborne nanoparticles than recycling of conventional plastics [246]. Meanwhile, the mechanical and rheological properties of most thermoplastic matrix nanocomposites remain unaffected after mechanical recycling, in spite of the polymer degradation caused by re-processing. Quite often, recycled samples outperform the pristine polymer matrix owing to the more homogeneous dispersion of the nanofiller, as well as to the improved intercalation/exfoliation of platelet-shaped nanoparticles such as clays [247].

The new frontier in “sustainable” composite materials is represented by self-reinforced polymer (SRP) composites, aka self-reinforced plastics, or single polymer composites. The key feature of these composites is that both the matrix and the reinforcement are thermoplastic materials having the same chemical composition, but different structure, because the reinforcement is a highly oriented version of the same polymer used for the matrix [248]. Since SRP composites are chemically homogenous, recycling is straightforward [249]. Meanwhile, they afford exceptional mechanical properties, such as high stiffness and high impact resistance [250]. However, (re-)manufacturing needs extra care, since the temperature must be controlled closely in order to selectively melt the matrix while keeping the reinforcement unaffected [248]. This is particularly problematic in FFF, because the polymer matrix should be heated and melted in the liquefier prior to being extruded through the print nozzle, but this would also melt the fibres. However, research is advancing in the field. Continuous fibre SRP composites were successfully printed after adding the fibres to the supercooled polymer matrix. This enabled a large processing window of nearly 50 °C, whereas the difference in melting temperature between the matrix and the (autologous) fibres was just 2 °C [251]. Mono-material fibre-reinforced composite constructs could also be printed by FFF harnessing the self-assembly of liquid-crystal polymer molecules into highly oriented domains during extrusion of the molten feedstock. These materials could also be recycled by re-melting and printing the polymer into new self-assembled structures [252]. In principle, FFF of thermotropic liquid crystal polymers can be combined with melt spinning-like effects by controlling the feed rate and the lateral velocity of the print head, thus producing all-fibre materials. The shift from multi-phase to mono-material composites ultimately holds the promise to facilitate recycling, while preserving all the advantages associated with the presence of a lightweight reinforcement [253].

As a general recommendation for mitigating the increased environmental footprint potentially associated with composite materials, the weight fraction of the filler should be kept as low as possible. At first, this may appear counter-intuitive because most properties of composite materials (their stiffness, for instance) are predicted to improve with higher filler loadings [254]. However, increasing the filler concentration promotes aggregation, heavily modifies the rheological behaviour of the polymer matrix, and engenders processing issues and printing faults, whose negative consequences ultimately outweigh the advantage of having more reinforcement [38]. Meanwhile, the properties of FFF parts should be commensurate with their intended application, rather than “maximized” [255], since overperformance, and hence overfilling, cause unnecessary environmental loads [12].

While technically feasible, recycling composite parts produced by FFF may pose additional challenges with respect to other composite systems due to the lack of dedicated standards. For example, most commercial products come with recycling codes that identify the materials out of which they are made, and often provide additional guidance for safe recycling and disposal. However, identification codes are not available for FFF parts, including composite ones. This may confuse the end-user, who is unaware of the real composition of the 3D printed object and hence unable to dispose of it properly for recycling [126,256]. This reminds us that “sustainability” has much to do with human behaviour and social practice, and not just with materials and technologies [257].

Ultimately, it is worth noting that the further adoption of recycling in polymer AM is strongly influenced by economic constraints, as well as by government regulations and incentives. It has been estimated that recycling PLA for FFF may be economically viable, given that the energy cost of shredding, drying and extruding is less than 1 USD/kg, whereas the cost of virgin PLA is around 17–18 USD/kg [135,258]. Similarly, the price of printable filaments produced with 80:20 (wt:wt) blends of recycled PET and recycled HDPE can be estimated to vary between 4 and 10 USD/kg, while virgin PETG was rated at 25 USD/kg, and virgin HDPE at almost 60 USD/kg [162]. Despite this potential economic advantage, the thermo-mechanical degradation of the polymer matrix and the recycling-induced deterioration of the reinforcement may lead to a noticeable functionality loss [50], which will finally lower the value (and hence the market price) of the produced filament. The assessment of the economic viability of recycling will thus depend on the nature of the constituent phases of the composite material, as well as on the specific procedure followed upon recycling [50]. Regardless of economic considerations, recycling may become the only viable option because of the stringent environmental legislation being enforced worldwide [135]. For example, the extended producer responsibility (EPR) scheme introduced in China in 2016 mandated the use of recycled and renewable raw materials in the manufacture of new products, where the stipulated proportions were set at 40% (on average) by 2020 for recycled materials, and at 20% by 2025 for renewable materials [259]. The circular economy represents a pillar of the growth strategy pursued by the European Union (EU), to the point that it is described as the trigger that will “modernise the EU industrial base to ensure its global competitive edge and preserve and restore the EU’s natural capital” [260]. Although the effectiveness of the EU policies and practices in the long term is still uncertain [261], it appears that the implementation of the Circular Economy Action Plan launched in 2015 has favoured the adoption of circular practices in numerous industries by inducing a strong technological push for sustainable growth, while also contributing to the creation of the required infrastructure. Notably, recycling has emerged in this context as the most widely used strategy for looping back materials into the manufacturing system [262].

## 7. Conclusions

Composite materials are essential for the growth of additive manufacturing in industrial settings. This is especially true for polymer-based technologies, like fused filament fabrication (FFF). The shift from conventional feedstock to composite materials may have negative consequences for the environment, though, because introducing a filler requires additional processing steps, and makes the disposal of end-of-life parts and print scraps more difficult. Recycling contributes substantially to mitigating the increased environmental footprint associated with composite feedstock, and offers substantial benefits:

- Being based on thermoplastic filaments, FFF may receive mechanically recycled plastics as the input material. Likewise, fillers can be recycled from other industrial activities, or sourced from agri-food waste. This will reduce the dependency on petrochemical products, and reduce the volume of landfilled materials;
- In spite of comprising heterogeneous phases, FFF composite parts can also be mechanically recycled like any other thermoplastic-matrix composites, which can be completed either through FFF (closed-loop recycling) or through other thermoplastic-based technologies (open-loop recycling). This will valorise end-of-life composite parts;
- While some challenges remain, such as the progressive degradation of the polymer matrix occurring upon thermo-mechanical (re-)processing, new strategies are emerging that will mitigate the functionality loss, such as the design of dedicated chain extenders that help restore the molecular weight of the polymer matrix;
- Research is being geared towards the development of new materials, like nanocomposites with minimal filler loading, that hold the promise to facilitate recycling and further lessen the environmental load of composite materials in FFF. A very promising area for future growth is represented by self-reinforced polymer composites, where the fibres and matrix have different structural organisation, but the same chemical composition. Although widening the processibility window of these materials represents a challenge in thermal processing methods like melt extrusion and FFF printing, the chemical homogeneity of self-reinforced polymer composites is a fundamental advantage for recycling over conventional multi-material composites.

## Figures and Tables

**Table 1:** Recycling-related keywords. References redirect to the literature source(s) from which each definition was taken.

Keyword	Definition	Ref.
Chemical recycling	Conversion from waste to new raw material through chemical means that convert the material (typically, a polymer) into smaller molecules to produce fuels and virgin plastic or plastic compounds.	[56]
Downcycling	The practice of downgrading the original material into a material of lesser quality. For example, producing rags from old clothing.	[54]
Life cycle assessment	Standardised analysis technique to assess environmental impacts associated with all the stages of a product’s life.	[55]
Material circularity indicator	For a given product, parameter measuring the extent to which the linear flow of material has been minimized and restorative flow maximized.	[57,58,59]
Mechanical recycling	Conversion from waste to new raw material through mechanical means like collection, sorting, washing, and grinding, which have minimal consequences to the material’s chemical composition. Steps may occur in a different order, repeatedly, or not at all.	[56]
Primary recycling	The recovered plastic is used in a new item having performance characteristics that are equivalent to virgin plastic. For example, PET recovered from post-consumer bottles being reused for producing new bottles. OR	[53]
Primary recycling	Recycling of post-industrial polymer waste (for example, obtained during injection or extrusion processes) to generate new products.	[61]
Quaternary recycling	Waste plastic is incinerated for producing (thermal) energy, and possibly sourcing residues as by-products. For example, tire-derived fuel.	[53]
Secondary recycling	The recovered plastic is used in a new item having inferior performance characteristics than virgin plastic. For example, PET recovered from post-consumer bottles being used for fibre spinning in textiles. OR	[53]
Secondary recycling	Recycling of post-consumer materials that are reprocessed into new products, which can be either higher-value products (upcycling) or lower-value products (downcycling).	[61]
Sustainability	Meeting the needs of the present without compromising the ability of future generations to meet their own needs.	[51]
Sustainability	The potential for something to continue indefinitely.	[33]
Sustainable materials	Materials used throughout our consumer and industrial economy that can be produced in required volumes without depleting non-renewable resources and without disrupting the established steady-state equilibrium of the environment and key natural resource systems.	[52]
Tertiary recycling	Waste plastic is used as the feedstock in a process that generates chemicals and fuels. For example, waste PET can be returned to diols and dimethyl terephthalate by chemical methods (glycolysis). Raw chemicals can then be used for making virgin PET.	[53]
Upcycling	The practice of refashioning something to a higher value. For example, a used plastic bottle is reinvented as a Moser light bulb [60].	[54]

## Data Availability

Data will be made available upon request.
